# A Systematic Review and Network Meta-analyses to Assess the Effectiveness of Human Immunodeficiency Virus (HIV) Self-testing Distribution Strategies

**DOI:** 10.1093/cid/ciab029

**Published:** 2021-01-20

**Authors:** Ingrid Eshun-Wilson, Muhammad S Jamil, T Charles Witzel, David V Glidded, Cheryl Johnson, Noelle Le Trouneau, Nathan Ford, Kathleen McGee, Chris Kemp, Stefan Baral, Sheree Schwartz, Elvin H Geng

**Affiliations:** 1 Washington University School of Medicine, St Louis, Missouri, USA; 2 Global HIV, Hepatitis and STI Programme, World Health Organization, Geneva, Switzerland; 3 Department of Public Health, Environments and Society, London School of Hygiene & Tropical Medicine, London, United Kingdom; 4 Department of Epidemiology, University of California, San Francisco, California, USA; 5 Department of Global Health and Development, London School of Hygiene and Tropical Medicine, London, United Kingdom; 6 Department of Epidemiology, John Hopkins School of Public Health, Baltimore, Maryland, USA

**Keywords:** HIV self-testing, network meta-analysis, systematic review, implementation

## Abstract

**Background:**

We conducted a systematic review and network meta-analysis to identify which human immunodeficiency virus (HIV) self-testing (HIVST) distribution strategies are most effective.

**Methods:**

We abstracted data from randomized controlled trials and observational studies published between 4 June 2006 and 4 June 2019.

**Results:**

We included 33 studies, yielding 6 HIVST distribution strategies. All distribution strategies increased testing uptake compared to standard testing: in sub-Saharan Africa, partner HIVST distribution ranked highest (78% probability); in North America, Asia, and the Pacific regions, web-based distribution ranked highest (93% probability), and facility based distribution ranked second in all settings. Across HIVST distribution strategies HIV positivity and linkage was similar to standard testing.

**Conclusions:**

A range of HIVST distribution strategies are effective in increasing HIV testing. HIVST distribution by sexual partners, web-based distribution, as well as health facility distribution strategies should be considered for implementation to expand the reach of HIV testing services.

## Teaser

We conducted a systematic review and network meta-analysis to assess effectiveness of human immunodeficiency virus self-test (HIVST) distribution strategies. HIVST distribution by sexual partners in sub-Saharan Africa and web-based in North America, Asia and the Pacific region resulted in the highest uptake.

Knowledge of human immunodeficiency virus (HIV) status is the gateway to HIV treatment and prevention services. The gap in HIV testing and diagnosis remains a critical barrier to meet global goals, particularly for certain populations including men, young people and key populations. HIV self-testing (HIVST), a World Health Organization (WHO)-recommended HIV testing approach, has been shown to be safe, accurate, and acceptable [1, 2]. It can be distributed using a range of strategies and can be used at the time and place of a tester’s choice, harnessing personal control, privacy, and convenience [3]. HIVST therefore has the potential to reach groups, communities, and individuals who face heightened barriers to accessing healthcare.

HIVST is currently being scaled up globally alongside other HIV testing approaches including traditional (standard) rapid HIV testing conducted by healthcare workers (HCWs) or trained lay providers at health facilities or in the community [4, 5]. HIVST distribution models need to be optimized for various settings, contexts, and populations to maximize impact [6]. Therefore, a single estimate of effect size that pools effects across distribution strategies, population types, and regions may mask useful information. Standard systematic reviews using pairwise meta-analyses can account for heterogeneity through subgrouping and meta-regression but remain constrained by the inability to compare multiple treatment arms and remain restricted to comparisons directly evaluated in primary studies.

Network meta-analyses offer a complementary methodology for comparing heterogenous implementation strategies: within networks, the effects of multiple interventions can be compared and direct comparisons can be used to generate indirect effect estimates; meta-regression can be utilized to account for heterogeneity, and distribution strategies can be ranked to identify which models are most effective [7]. We therefore conducted pairwise and network meta-analyses to compare the effects of a variety of HIVST distribution strategies on HIV testing uptake, positivity yield, and linkage to inform HIVST implementation.

## METHODS

### Search Strategy and Selection Criteria

We searched MEDLINE, EMBASE, Cochrane, Web of Science, Global Health, Social Policy and Practice, Health Management Information Consortium, EBSCO, CINAHL Plus, Sociological Abstracts, and PsycINFO databases for randomized controlled trials (RCTs) conducted between 1 January 2006 and 4 June 2019, with additional searches of clinical trial registries, and major HIV conferences up until 31 July 2019 [8]. Abstracts were then screened and reviewed for eligibility by 2 authors. We included randomized controlled trials and observational studies that compared a HIVST distribution strategy with any other HIVST distribution or HIV testing strategy, from all settings and population groups, and reporting HIV testing uptake, HIV positivity, or linkage. Data from included studies were abstracted by one author into a commercially available web-based relational database tool (https://airtable.com/), reviewed by a second author, and discrepancies were resolved by a third author. Risk of bias in randomized controlled trials was assessed across 5 domains according to the Cochrane risk of bias tool [9] and for observational studies using the Newcastle Ottawa risk of bias tool [10].

### Data Analysis

We conducted and reported analyses according to Preferred Reporting Items for Systematic Reviews and Meta-Analyses (PRISMA) guidelines for pairwise and network meta-analyses (NMAs) [11, 12]. We grouped HIV testing approaches according to test distribution location (at a healthcare facility or in the community), who dispensed the test (peer, partner, or HCW) and what test was used (HIVST or traditional HIV test). This approach yielded 6 delivery strategies (Table 1, Supplementary Appendix 1). For analysis, we incorporated numerators and denominators from individually randomized trials and cluster-adjusted relative effect estimates for cluster RCTs (where not applicable the Cochrane design effect was applied) [9]. We first conducted pairwise meta-analysis using generic inverse variance methods to generate risk ratios (RR) with 95% confidence intervals (CI) and then conducted a network meta-analysis if there were sufficient studies contributing to distribution strategies (detailed methods in Supplementary Appendix 1).

**Table 1.** HIV TestingStrategies in Included Studies

HIV Testing Strategies	Short Term
Partner HIVST distribution to sexual partner in community	Partner-community-HIVST
Peer distribution of HIVST in community	Peer-community-HIVST
HIVST distribution by online ordering and mail distribution	Online-mail-HIVST
HCW distribution of HIVST at facility^a^	HCW-facility-HIVST
HCW distribution of HIVST in community	HCW-community-HIVST
Vending machine HIVST distribution in community	Vending-community-HIVST
HCW administration of traditional HIV test in community	HCW-community-TT
HCW administration of facility-based traditional HIV test	HCW-facility-TT

Abbreviations: HCW, healthcare worker; HIV, human immunodeficiency virus; HIVST, oral HIV self-test; TT, traditional HIV test (finger-prick rapid test performed by HCW).

^a^Includes both HIVST distributed and conducted at the health facility and HIVST distributed at the facility to conduct elsewhere.

To address intransitivity in the network meta-analysis (the violation of the assumption that different sets of randomized trials are similar, on average, in all important factors other than the intervention comparison being made [13]), 2 networks were developed, one for Sub-Saharan Africa and another for North America, Asia, and Pacific region, primarily because distribution strategies and population groups differed substantially between these settings. We used random effects logit models to account for the heterogeneity of treatment effects across studies in the networks and selected final models by evaluating a combination of the deviance information criterion (DIC), Markov chain Monte Carlo (MCMC) error and trace and density plots [14]. We present risk ratios (RR) with 95% credible intervals (CrI) for network meta-analyses. We additionally evaluated inconsistency between direct and indirect comparisons for closed loop network estimates using the node-splitting technique. Results are presented in relative effects tables and forest plots. Ranking probabilities (the probability that a distribution strategy is selected as the best, second best, etc) are displayed using ranking plots, where a ranking probability of 1 (100%) represents the highest ranking of a distribution strategy and 0 the lowest.

To explore the heterogeneity of population types included in the network and the impact of this on network estimates, we conducted sensitivity analyses where female sex-workers were excluded (we hypothesized that this key population group may respond to testing strategies differently to general and other key populations) and conducted metaregression by gender group. The meta and gemtc packages in R programming software were used for all analyses [15].

## RESULTS

**Figure 1.**
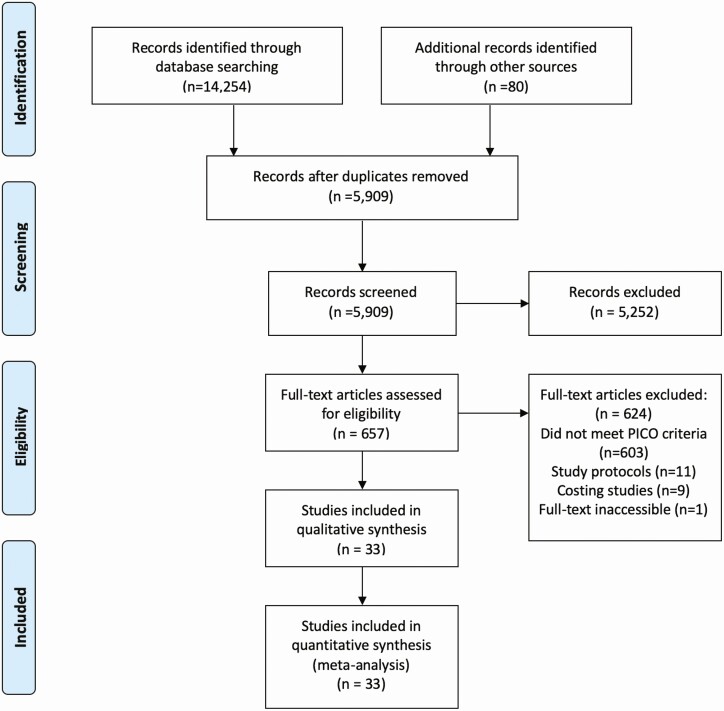
PRISMA diagram. Abbreviation: PICO, population, intervention, comparison and outcome; PRISMA, Preferred Reporting Items for Systematic Reviews and Meta-Analyses.

Searches yielded 14 254 citations of which 24 RCTs and 9 observational studies were included in the review (Figure 1). Characteristics of included studies and interventions are presented in Table 2A and Table 2B. Further intervention characteristics and outcome definitions are presented in the supplementary materials (Supplementary Table 1 and Supplementary Tables 2A–2C).

**Table 2A.** Included Study Characteristics: Sub-Saharan Africa

Study/Year	Country	Sample Size^a^	HIVST Distribution Strategy	Population	Source	Study Design
Pai 2018	South Africa	2500	HCW at health facility	General population	Conference presentation	Cohort
Kelvin 2019b	Kenya	2196	HCW at health facility	FSW	Journal article	RCT
Kelvin 2018	Kenya	549	HCW at health facility	Migrant/mobile men	Journal article	RCT
Kelvin 2019a	Kenya	2262	HCW at health facility	Migrant/mobile men	Journal article	RCT
Dovel 2018	Malawi	5885	HCW at health facility	General population	Conference presentation	Cluster RCT
Pettifor 2018	South Africa	284	HCW at health facility	Women (18–24 y)	Conference presentation	RCT
Indravudh 2018	Malawi	3457	HCW in community	General population	Conference presentation	Cluster RCT
Indravudh 2019	Malawi	7880	HCW in community	General population	Conference presentation	Cluster RCT
Mulubwa 2019^**b**^	Zambia	26 973	HCW in community	General population	Journal article	Cluster RCT
Tsamwa 2018	Zambia	5005	HCW in community	General population	Conference presentation	Cluster RCT
Nichols 2019	Zambia	12 081	HCW in community	Young women (16–24 y)	Conference presentation	Cohort
Gichangi 2018	Kenya	1410	Partner distribution in community	Male partners of ANC	Journal article	RCT
Masters 2016	Kenya	600	Partner distribution in community	Male partners of ANC	Journal article	RCT
Choko 2019a	Malawi	2349	Partner distribution in community	Male partners of ANC	Journal article	Cluster RCT
Choko 2019b (ii)	Malawi	7814	Partner distribution in community	Male partners of ANC	Conference presentation	Cluster RCT
Choko 2019b (i)	Malawi	5054	Partner distribution in community	Partners of HIV positive	Conference presentation	Cluster RCT
Dovel 2019	Malawi	484	Partner distribution in community	Partners of HIV positive	Conference presentation	RCT
Van Der Elst 2017	Kenya	1027	Peers in community	MSM	Conference presentation	Cohort
Ortblad 2017	Uganda	960	HCW at health facility / peer in community	FSW	Journal article	Cluster RCT
Chanda 2017	Zambia	965	HCW at health facility / peer in community	FSW	Journal article	Cluster RCT

Abbreviations: ANC, antenatal client; FSW, female sex workers; HCW, healthcare worker; HIV, human immunodeficiency virus; MSM, men who have sex with men; RCT, randomized controlled trial.

^**a**^Sample size for RCTs represents number randomized to offer of HIV testing, for cohorts represents number tested for HIV.

^**b**^Comparison arm was community-based tradition rapid antiretroviral therapy (ART) testing by health care worker.

**Table 2B.** Included Study Characteristics: North America, Asia, and the Pacific Region

Study/Year	Country	Sample Size^a^	HIVST Distribution Strategy	Population	Source	Study Design
Patel 2018	USA	100	HCW at health facility	ER HIV test decliners	Journal article	RCT
Katz 2018	USA	230	HCW at health facility	MSM and TGW	Journal article	RCT
Jamil 2017	Australia	362	HCW at health facility	MSM and TGW	Journal article	RCT
MacGowan 2019	USA	2665	Online ordering and mail	MSM and TGW	Journal article	RCT
Wray 2018	USA	65	Online ordering and mail	MSM and TGW	Journal article	RCT
Merchant 2018	USA	425	Online ordering and mail	MSM (18–24yrs)	Journal article	RCT
Stafylis 2018^**c**^	USA	1134	Vending machine at sex work venue	Clients of FSWs	Journal article	Cohort
Qin 2016	China	1189	Online ordering and mail	MSM and TGW	Journal article	Cohort
Nguyen 2019	Thailand	3978	Peers in community	Key populations^**a**^	Journal article	Cohort
Tang 2018	China	1381	Online ordering and mail	MSM and TGW	Journal article	Cluster RCT (SW)
Phanuphak 2018^**c**^	Thailand	571	Online ordering and mail	MSM and TGW	Journal article	Cohort
Lightfoot 2018	USA	165	Peers in community	MSM and TGW	Journal article	Cohort
Rich 2018^**c**^	New Zealand	498	Online ordering and mail	MSM and TGW	Conference presentation	Cohort
Green 2018	Vietnam	1351	HCW in community	MSM and TGW	Journal article	Cohort
Wang 2017	Hong Kong, SAR China	430	Online ordering and mail	MSM and TGW	Journal article	RCT

Abbreviations: ER, emergency room; FSW, female sex workers; HCW, healthcare worker; HIV, human immunodeficiency virus; HIVST, oral HIV self-test; MSM, men who have sex with men; RCT, randomized controlled trial; SW, Stepped Wedge; TGW, transgender women.

^**a**^Sample size for RCTs represents number randomized to offer of HIV testing, and for cohorts this represents number tested for HIV.

^**b**^Includes 55% MSM, 39% persons who inject drugs (PWID).

^**c**^Comparison arm was community-based tradition rapid antiretroviral therapy (ART) testing by healthcare worker.

Nineteen studies were conducted in sub-Saharan Africa: Malawi [16–20], Kenya [21–25], South Africa [26, 27], Uganda [28], and Zambia [29–31], 7 in the United States [32–38], 2 in China [39, 40], 2 in Vietnam [41, 42] and 1 each in Hong Kong [43], New Zealand [44], and Australia [45]. Studies conducted in sub-Saharan Africa (Table 2A) were focused on the male partners of women attending antenatal clinics (N = 4), partners of HIV-positive people on antiretroviral therapy (ART) (index clients) (N = 2), general populations (N = 5), young women (N = 1), female sex workers (FSWs) (N = 3), truck drivers (N = 2), and men who have sex with men (MSM) (N = 1). In North America, Asia, and the Pacific region (Table 2B), the study populations included MSM and transgender women (TGW) (N = 12), patients declining HIV testing in an emergency department (N = 1), clients of FSWs (N = 1), and 1 study included MSM, persons who inject drugs (PWID), and other key population groups. Most studies compared HIVST delivery to standard HCW-administered facility based rapid HIV tests (traditional HIV testing), and 4 compared HIVST delivery to community based traditional rapid HIV testing administered by HCWs. HIV self-tests were delivered through HCW distribution in the health facility (N = 11) or community (N = 6), web-based ordering and mail delivery (N = 8), partners (N = 6), peers (N = 5), and vending machines (N = 1).

All RCTs were judged as high risk of bias primarily due to self-reported outcomes and lack of blinding of patients and study personnel, as well as lack of blinding of outcome assessors (Supplementary Table 2A). The majority of observational studies were judged as poor or fair quality predominantly due to selection of comparison arms, which were not truly representative of the intervention arms and underreporting of ascertainment of exposure (Supplementary Table 2B).

### Effects of HIVST Distribution Strategy on Uptake of HIV Testing

#### Uptake in Sub-Saharan Africa

**Figure 2.**
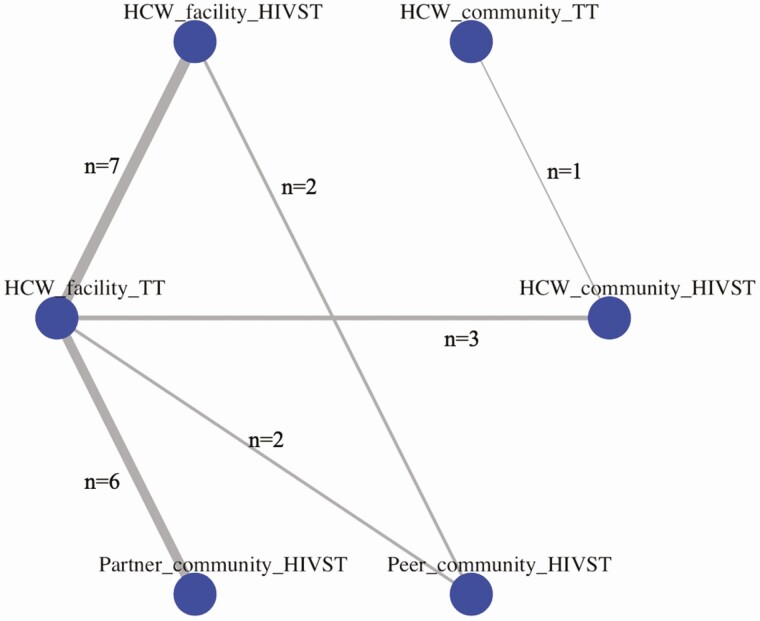
Sub-Saharan Africa network map: uptake of HIV testing. Network map represents the number of studies contributing to the direct comparisons in the network. Abbreviations: HCW, healthcare worker; HIV, human immunodeficiency virus; HIVST, HIV self-testing; TT, traditional HIV test.

**Figure 3.**
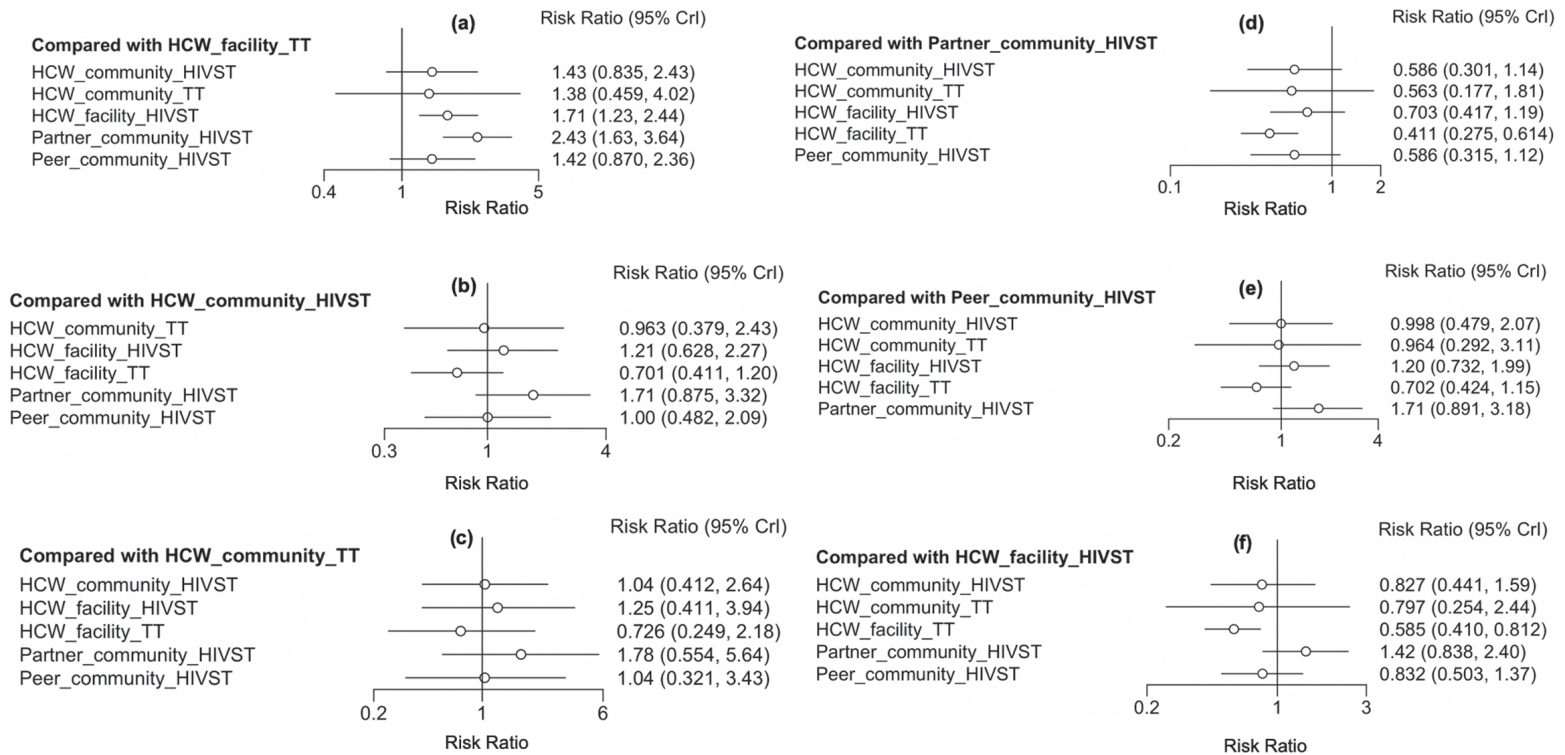
Sub-Saharan Africa network estimates of HIV testing uptake. Abbreviations: CrI, credible interval; HCW, healthcare worker; HIV, human immunodeficiency virus; HIVST, HIV self-testing; TT, traditional HIV test.

Six direct comparisons contributed to this network meta-analysis (Figure 2), with the largest number of studies (7 studies) comparing HCW HIVST distribution at the health facility (HCW-facility-HIVST) to HCW administration of traditional rapid HIV tests at the health facility (HCW-facility-traditional HIV test [HCW-facility-TT]), followed by the comparison of partner community HIVST distribution (partner-community-HIVST) with HCW administration of traditional HIV tests at the health facility (HCW-facility-TT) (6 studies). Network estimates showed that partner (RR 2.43, 95% CrI: 1.63–3.64) and facility-based HIVST (RR 1.71, 95% CrI: 1.23–2.44) distribution methods resulted in higher HIV testing service (HTS) uptake than traditional HCW facility-based testing (Figure 3A), and there was some evidence that partner distribution may increase uptake more than peer distribution methods (RR 1.71, 95% CrI: .89–3.18) (Figure 3, Supplementary Table 4).

**Figure 4.**
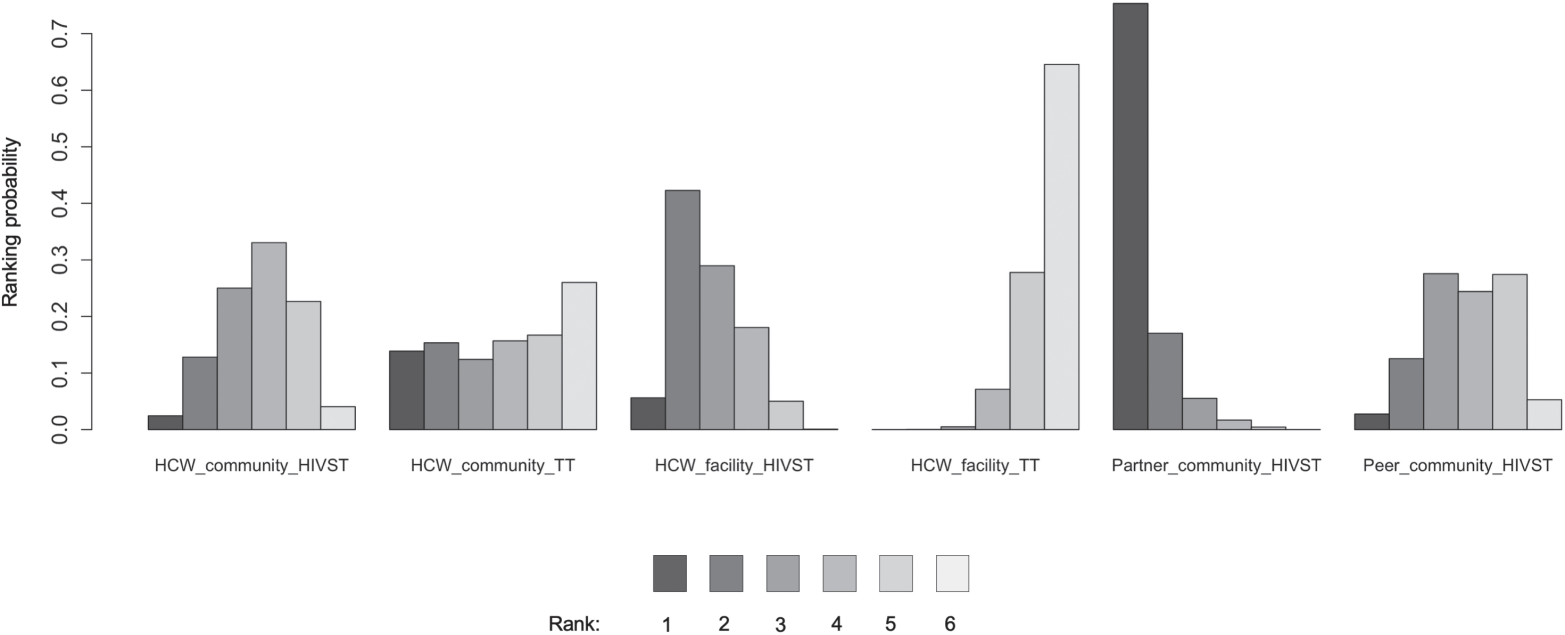
HIV testing strategies ranking probabilities for HIV testing uptake. For each strategy the colored bars represent the probability that that strategy ranks first, second, third, and so forth. Darker colors represent high ranking (most effective); light colors represent low ranking (least effective). Abbreviations: HCW, healthcare worker; HIV, human immunodeficiency virus; HIVST, HIV self-testing; TT, traditional HIV test.

Ranking probabilities (Figure 4) demonstrated that HIV testing uptake was highest with partner HIVST distribution in the community (ranked highest uptake in 78% of simulations) or HCW HIVST distribution at a health facility (ranked second in 45% of simulations), and that all HIVST models had higher HIV testing uptake than HCW facility based traditional HIV testing, which ranked lowest in 66% of simulations. This is supported by data from the pairwise meta-analysis of risk differences, which showed 90% (95% CI: 63–100%) higher uptake from partner HIVST distribution compared to traditional facility based HIV testing and 50% (95% CI: 29–73%) increase in HIV testing uptake when HIVST was distributed at a health facility compared to traditional facility-based HIV testing (Supplementary Table 5).

We conducted meta-regression including population type as a covariate (Supplementary Table 6), After adjustment, partner HIVST distribution continued to have the strongest effect on HIV testing uptake compared to HCW traditional HIV testing at health facilities (RR 1.82, 95% CrI: 1.19–21.58). We additionally conducted a sensitivity analysis where the NMA was conducted after exclusion of FSWs. In this analysis, partner HIVST distribution (RR 2.39, 95% CrI: 1.59–3.64) and facility-based HIVST distribution (RR 2.12, 95% CrI: 1.35–3.34) continued to have the strongest effect on HIV testing uptake (Supplementary Table 7).

#### Uptake in North America, Asia, and Pacific Network

**Figure 5.**
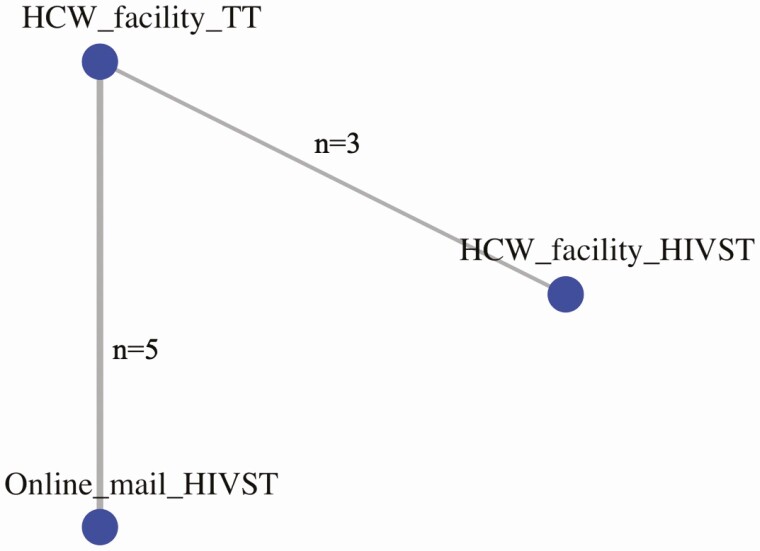
North America, Asia, and Pacific network and comparisons: uptake of HIV testing. Network map represents the number of studies contributing to the direct comparisons in the network. Abbreviations: HCW, healthcare worker; HIV, human immunodeficiency virus; HIVST, HIV self-testing; TT, traditional HIV test.

**Figure 6.**
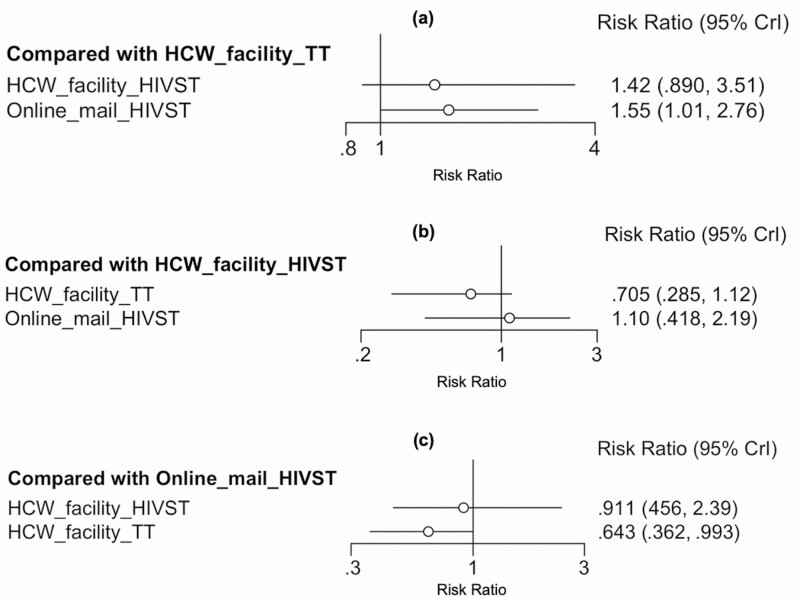
North America, Asia, and Pacific network relative effects. Abbreviations: CrI, credible interval; HIV, human immunodeficiency virus; HIVST, HIV self-testing; TT, traditional HIV test.

**Figure 7.**
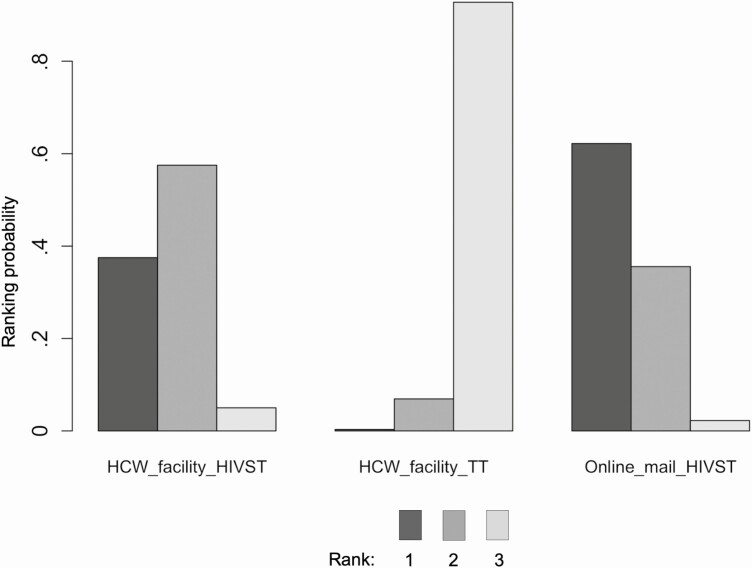
North America, Asia, and Pacific ranking probabilities. For each strategy the coloured bars represent the probability that that strategy ranks first, second, third, and so forth. Darker colors represent high ranking (most effective); light colors represent low ranking (least effective). Abbreviations: HIVST, human immunodeficiency virus self-testing; TT, traditional HIV test.

Eight studies and 3 HIVST distribution strategies contributed to the network meta-analysis of uptake of HIV testing (Figure 5) in North America, Asia, and the Pacific region. Network estimates (Figure 6, Supplementary Table 8) showed web-based ordering of HIVST with subsequent delivery by mail to be the most effective strategy to improve testing uptake compared to traditional facility based HIV testing (RR 1.55, 95% CrI: 1.01–2.76) (Figure 6A), and both HIVST distributions strategies (web-based ordering and facility distribution) ranked higher than traditional HIV testing (Figure 7, Supplementary Table 7). In pair-wise meta-analysis, web-based ordering and mail HIVST distribution resulted in 39% (95% CI: 27–52%) increase in HIV testing uptake compared to traditional HIV testing at a health facility (Supplementary Table 5). The majority of these studies were conducted among MSM and TGW populations, making these data most relevant to this population group.

### Effect of HIVST Distribution Strategy on HIV Positivity Among Those Tested for HIV

#### Positivity in Sub-Saharan Africa

Six direct comparisons contributed to pairwise meta-analysis of the effect of distribution strategy on HIV positivity in Sub-Saharan Africa (Table 3). Distribution strategies assessed in RCTs showed variable results, with wide confidence intervals including no difference in positivity for all comparisons. Cohort studies had overall larger samples of individuals tested for HIV and showed higher HIV positivity with HIVST distribution in a few instances: One cohort study conducted in South Africa [26] showed higher positivity rates with facility HIVST distribution compared to routine facility based HIV testing in the general community (RR 1.50; 95% CI: 1.14–1.97). A further cohort study from Kenya conducted in MSM showed increased positivity rates with peer distribution of HIVST compared with facility distribution (RR 2.47; 95% CI: 1.46–4.18) [25]. Another cohort study conducted among youth in Zambia showed lower positivity rates with HCW community distribution than with routine facility-based HIV testing by HCWs (.33; 95% CI: .12–.88) [46].

**Table 3.** HIV Positivity Among Tested: by Distribution Strategy, Region, Study Design and Population Subgroup

Region	Strategy 1	Strategy 2	Design	Population Type	Pooled Risk Ratio	Studies
Sub-Saharan Africa	Partner -community- HIVST	HCW-facility-TT	RCT	Male partners of ANC	.58 (.18–1.87)	Choko 2019b, Masters 2016, Choko 2019a
				Partners of HIV positive	1.42 (.74–2.71)	Choko 2019b, Dovel 2019
	HCW-facility - HIVST	HCW-facility-TT	RCT	FSW	.79 (.58–1.08)	Chanda 2017, Ortblad 2017, Kelvin 2019b
				Truck drivers	1.02 (.05–20.7)	Kelvin 2018, Kelvin 2019a
				General population	.70 (.20–2.45)	Dovel 2018
			Cohort	General population	1.50 (1.14–1.97)	Pai 2018
	Peer -community- HIVST	HCW-facility-TT	RCT	FSW	.92 (.72–1.18)	Chanda 2017, Ortblad 2017
			Cohort	MSM and TGW	2.47 (1.46–4.18)	Van Der Elst 2017
	Peer - HIVST	HCW-facility-HIVST	RCT	FSW	.78 (.48–1.28)	Chanda 2017, Ortblad 2017
	HCW community - HIVST	HCW-facility-TT	Cohort	Youth	.33 (.12–.88)	Nichols 2019
	HCW-community - HIVST	HCW-community-TT	RCT	General population	.94 (.78–1.14)	Mulubwa 2019
North America, Asia, Pacific	HCW-facility - HIVST	HCW-facility-TT	RCT	MSM and TGW	2.00 (.44–9.11)	Jamil 2017, Katz 2018
	Peer -community- HIVST	HCW-facility-TT	Cohort	MSM and TGW	2.15 (.71–6.56)	Lightfoot 2018, Nguyen 2019*
	Online and mail – HIVST	HCW community-TT	Cohort	MSM and TGW	2.86 (1.23–6.65)	Rich 2018, Phanuphak 2018
	Online and mail - HIVST	HCW-facility-TT	RCT	MSM and TGW	1.36 (.71–2.63)	Wray 2018, Merchant 2018, MacGowan 2019, Wang 2017
			Cohort	MSM and TGW	1.44 (.77–2.69)	Qin 2017
	Vending - HIVST	HCW-community-TT	Cohort	FSW clients	1.19 (.51–2.79)	Stafylis 2018

Abbreviations: ANC, antenatal client; FSW, female sex workers; HCW, healthcare worker; HIV, human immunodeficiency virus; HIVST, oral HIV self-test; MSM, men who have sex with men; RCT, randomized controlled trial; TT, traditional HIV test (finger-prick rapid test performed by HCW).

*55% were MSM, remaining population included PWID (39%) and others (6%).

#### Positivity in North America, Asia, and the Pacific Region

Five direct comparisons contributed to pairwise meta-analysis of the effect of HIV distribution strategy on HIV positivity in North America, Asia, and the Pacific region (Table 3). All (except one) studies were conducted among MSM and TGW in these settings; all analyses showed higher positivity with HIVST distribution strategies, although this only reached statistical significance for the comparison of online ordering and mail distribution versus HCW community-based traditional HIV testing, based on cohort data from Thailand and New Zealand (RR 2.86; 95% CI: 1.23–6.65) [41, 44].

### Effect of Distribution Strategy on Linkage to ART or HIV Care Among HIV Positive

#### Linkage in Sub-Saharan Africa

Six direct comparisons contributed to pairwise meta-analysis of the effect of distribution strategy on linkage to ART or HIV care among HIV positive people in Sub-Saharan Africa (Table 4). There appeared to be no difference in linkage when individual HIVST distribution strategies were compared to traditional HIV testing by HCWs at the health facility or in the community (risk ratios and 95% CIs are presented in Table 4).

**Table 4.** Linkage to ART or Any Care Among HIV Positive by Distribution Strategy, Region, Study Design and Population Subgroup

Region	Strategy 1	Strategy 2	Design	Population Type	Pooled Risk Ratio	Studies
Sub-Saharan Africa	Partner-community-HIVST	HCW-facility-TT	RCT	Male partners of ANC clients	.95 (.56–1.59)	Choko 2019b, Masters 2016, Choko 2019a
				Partners of HIV positive	.62 (.19–1.99)	Choko 2019b, Dovel 2019
	HCW-facility-HIVST	HCW-facility-TT	RCT	FSW	.83 (.66–1.06)	Chanda 2017, Ortblad 2017, Kelvin 2019b
				General population	.84 (.55–1.30)	Dovel 2018
	Peer-community-HIVST	HCW-facility-TT	RCT	FSW	.83 (.63–1.09)	Chanda 2017, Ortblad 2017
			Cohort	MSM and TGW	.99 (.78–1.27)	Van Der Elst 2017
	Peer-community-HIVST	HCW-facility-HIVST	RCT	FSW	1.05 (.73–1.49)	Chanda 2017, Ortblad 2017
	HCW-community-HIVST	HCW-facility-TT	RCT	General population	.96 (.76–1.21)	Tsamwa 2018
North America, Asia, Pacific	HCW-facility-HIVST	HCW-facility-TT	RCT	MSM and TGW	1.10 (.60–2.00)	Jamil 2017, Katz 2018
	Online and mail-HIVST	HCW-community-TT	Cohort	MSM and TGW	.87 (.54–1.38)	Rich 2018
	Online and mail-HIVST	HCW-facility-TT	RCT	MSM and TGW	.72 (.51–1.01)	MacGowan 2019, Wang 2017
	Vending-HIVST	HCW-community-TT	Cohort	FSW clients	.65 (.41–1.03)	Stafylis 2018

Abbreviations: ANC, antenatal client; ART, antiretroviral therapy; FSW, female sex workers; HCW, healthcare worker; HIV, human immunodeficiency virus; HIVST, oral HIV self-test; MSM, men who have sex with men; RCT, randomized controlled trial; TGW, transgender women; TT, traditional HIV test (finger-prick rapid test performed by HCW).

#### Linkage in North America, Asia and the Pacific Region

Four direct comparisons and 3 HIVST distribution strategies contributed to pairwise meta-analysis of the effect of HIV distribution strategy on linkage in North America, Asia, and the Pacific region (Table 4). Similarly, there appeared to be no difference in linkage between HIVST distribution strategies and traditional HIV testing at the health facility or in the community (risk ratios and 95% CIs are presented in Table 4).

## DISCUSSION

All HIVST distribution strategies showed higher HIV testing uptake than traditional facility-based health worker administered HIV tests. The network meta-analyses revealed that across sub-Saharan Africa, secondary HIVST distribution through sexual partners (most commonly to male partners of antenatal clients) resulted in the highest uptake of HIV testing compared to all other HIVST distribution strategies, including HCW facility-based, HCW community-based and secondary distribution by peers. Across North America, Asia, and the Pacific region, where studies primarily focused on MSM and TGW, web-based tools with subsequent mail delivery of HIVSTs showed the highest uptake compared to facility-based HIVST distribution by HCWs. HIVST distribution by HCWs at health facilities ranked second in all settings. In pairwise meta-analyses, we found that there was little or no difference in HIV positivity or linkage with HIVST distribution strategies compared to standard testing across regions and populations.

Secondary distribution strategies by partners or peers can leverage existing sexual and social networks to access marginalized groups [47–50]. The high testing uptake resulting from partner distribution by antenatal clients suggests that this strategy can have a substantial impact on increasing testing in men who do not routinely attend health services. Peer distribution strategies were predominantly explored in studies of FSWs, determinants specific to sex workers, such as criminalization and stigma means that these may not reflect the effectiveness of peer distribution strategies among other populations in sub-Saharan Africa [51]. Future research should explore the use of peer HIVST distribution to other at-risk peer network groups (eg, MSM) and consider expanding distribution strategies for FSWs [48, 52].

In North America, Asia, and the Pacific region, our review found that web-based mail delivery of HIVST to primarily MSM and TGW populations was commonly used and improved uptake of HIV testing. The success of this intervention suggests that convenience and confidentiality—which are identified desirable features of self tests [1, 6]—are valued by those who may not otherwise access testing. Further research on HIVST distribution strategies for MSM and TGW in settings where web-based mail distribution is not feasible will be needed to inform approaches for this group in less well-resourced areas.

We found that even when offered by HCWs in healthcare facilities, HIVST can increase HIV testing. Although this approach is less focused on reaching underserved groups who do not routinely attend health facilities, the fact that this strategy showed better uptake than traditional health worker administered facility-based testing across a wide variety of population groups implies that in routine service delivery settings, providing the option of a different testing modality can increase testing and enhance reach among those who do attend health facilities but do not routinely test.

The effects of community based HIVST distribution campaigns on HIV testing uptake were modest in comparison to other distribution strategies: 4 studies from Malawi and Zambia employed lay health workers and community volunteers to distribute HIVST in general communities; these strategies showed lower uptake than others, possibly indicating already well established HIV testing programs and high coverage of testing and treatment. These studies did, however, show benefits in frequently missed subgroups such as men and young people [19, 29, 53], indicating that community distribution strategies should be focused on subpopulations that have greatest gaps in testing coverage.

The effects of individual distribution strategies on HIV positivity rates varied by study design, with all RCTs showing no difference in positivity rates between HIVST distribution strategies and traditional HIV testing, and cohort studies showing either no difference or higher positivity rates in a few instances. Similarly, compared to traditional HIV testing by HCWs, there appeared to be no difference in linkage between HIVST and traditional HIV testing by HCWs in the health facility or community.

This analysis was limited by overall few studies contributing to each strategy, resulting in weak networks in the network meta-analysis and insufficient data to draw conclusions on optimum strategies for increasing HIV positivity rates and linkage in pairwise comparisons. In addition, we included unadjusted estimates from observational studies. Despite these limitations, the use of an NMA approach allowed for comparisons across strategies to increase uptake that were not directly assessed by pair-wise meta-analysis. Although NMAs have traditionally been applied to clinical drug efficacy trials, this analysis shows that the utility of these methods extends to implementation strategies if accompanied by careful examination of heterogeneity.

In this review a range of HIVST distribution models were found to be effective in increasing HIV testing uptake and achieve positivity and linkage similar to standard HIV testing methods. Promising models include secondary distribution of HIV self-tests through sexual partners of HIV-positive individuals and clinic attendees in sub-Saharan Africa and web-based mail distribution to MSM in North America, Asia, and the Pacific region. Facility-based HIVST distribution may additionally be considered to improve efficiency and testing coverage in health facilities.

## Supplementary Data

Supplementary materials are available at *Clinical Infectious Diseases* online. Consisting of data provided by the authors to benefit the reader, the posted materials are not copyedited and are the sole responsibility of the authors, so questions or comments should be addressed to the corresponding author.

ciab029_suppl_Supplementary_Materials

## Notes

***Financial support.*** National Institutes of Health (grant numbers: K24AI134413 and KL2TR002346-04), the Bill and Melinda Gates Foundation (grant number OPP1215984), National Institute for Health Research (NIHR) (grant number PG-1212 -20006), and ViiV Healthcare.

***Potential conflicts of interest.*** T. C. W. reports that their salary is grant funded by NIHR. All other authors report no potential conflicts. All authors have submitted the ICMJE Form for Disclosure of Potential Conflicts of Interest. Conflicts that the editors consider relevant to the content of the manuscript have been disclosed.
