# Supplementary material for: A Systematic Review and Network Meta-analyses to Assess the Effectiveness of Human Immunodeficiency Virus (HIV) Self-testing Distribution Strategies
Source: Clin Infect Dis. 2021 Jan 20;73(4):e1018–28. doi: 10.1093/cid/ciab029 (PMC8366833; doi:10.1093/cid/ciab029)
Supplement: ciab029_suppl_Supplementary_Materials [file ciab029_suppl_supplementary_materials.docx]

Table of Contents

[S1 Table: HIVST distribution strategy details 2](#_Toc58527437)

[S2(a)Table: Risk of bias for included studies - RCT 7](#_Toc58527438)

[S2(b)Table: Risk of bias for included studies – Cohorts 8](#_Toc58527439)

[S3(a)Table: HIV testing uptake among all randomized or enrolled - outcomes extracted from contributing studies 9](#_Toc58527440)

[S3(b) Table: HIV positivity among HIV tested – outcomes extracted from contributing studies 11](#_Toc58527441)

[S3(c) Table: Linkage to ART or HIV care among HIV positive: outcomes extracted from contributing studies 14](#_Toc58527442)

[S4 Table: Direct pairwise meta-analyses of effects of HIVST distribution strategies on HIV testing uptake 16](#_Toc58527443)

[S5 Table: Sub-Saharan Africa network meta-analysis relative effects (league) table of HIVST distribution strategies - HIV testing uptake 17](#_Toc58527444)

[S6 Table: Sub-Saharan Africa network meta-analysis relative effects (league) table, metaregression (by population type) - HIV testing uptake 17](#_Toc58527445)

[S7 Table: Sub-Saharan Africa network meta-analysis – sensitivity analysis (FSW excluded), HIV testing uptake 18](#_Toc58527446)

[S7(a) League table 18](#_Toc58527447)

[S7(b) Network meta-analysis ranking probabilities 18](#_Toc58527448)

[S8 Table: North America; Asia & Pacific region network meta-analysis relative effects (league) table – HIV testing uptake 19](#_Toc58527449)

[S1 Appendix: Supplementary methods 20](#_Toc58527450)

## S1 Table: HIVST distribution strategy details

| **Study/Year** | **HIVST Delivery Method** | **Who distributed HIVST** | **Where HIVST delivered** | **How HIVST delivered** | **Who administered HIVST** | **Testing choice offered?** | **Testing support** | **Additional intervention components** | **Comparison** |
| --- | --- | --- | --- | --- | --- | --- | --- | --- | --- |
| **Chanda 2017** | Facility & secondary by peer | HCW or peer | Facility and Community/ home | In person | Participant | No | Group demonstration | 2 HIVST arms: HIVST coupon for facility testing or HIVST delivered to participants by peer | Routine facility-based HIV testing |
| **Choko 2019a*** | Secondary | HCW to ANC client to partner | Facility and Community/ home | In person | Participant | No | Instruction enhancement, video or study hotline | 5 HIVST arms: HIVST only; HIVST+$3; HIVST +$10; HIVST + reminder; HIVST +lottery (Incentives were given to the men) | Routine facility-based HIV testing |
| **Choko 2019b (i)** | Secondary | HCW to ART client to partner | Facility and Community/ home | In person | Participant | No | Instruction enhancement, video or study hotline | 2 HIVST arms: HIVST only; HIVST+$10 incentive (Incentives were given to the partners) | Routine facility-based HIV testing |
| **Choko 2019b (ii)** | Secondary | HCW to ANC client to partner | Facility and Community/ home | In person | Participant | No | Instruction enhancement, video or study hotline | 2 HIVST arms: HIVST only; HIVST+$10 incentive (Incentives were given to the men, conditional on attendance of HIV treatment or prevention services within 28 days.) | Routine facility-based HIV testing |
| **Dovel 2018** | Facility-based | HCW | Clinic outpatient waiting room | In person | Participant or assisted by HCW | No | Group demonstration | Trained counselor available for post-test counselling. Group test demonstration in waiting room. | Routine facility-based HIV testing |
| **Dovel 2019** | Secondary | HCW to ANC client to partner | Facility and Community/ home | In person | Participant | No | Instruction enhancement, video or study hotline | Passive referral slips for confirmatory testing at facility; disclosure counseling; map to nearest facility | Routine facility-based HIV testing |
| **Gichangi 2018** | Secondary | HCW to ANC client to partner | Facility and Community/ home | In person | Participant | No | Instruction enhancement, video or study hotline | Advise on partner negotiation and communication | Invitation card to partner to attend health services for routine or HIV care |
| **Green 2018** | Community-based | HCW | Community-based organization | In person | Participant or assisted by HCW | Yes: Supervised or unsupervised | In−person observation or supervision | Clients could choose between either blood-based or oral fluid self-test. Supported confirmatory testing and counselling for reactive clients. | Routine facility-based  HIV testing |
| **Indravudh 2018** | Community-based | Community volunteers | In community door-to-door | In person | Participant | No | In−person demonstration or training | HIVST distributers remunerated per HIVST distributed; post-test support provided | Routine facility-based HIV testing |
| **Indravudh 2019** | HCW community-based | Community volunteers | In community door-to-door | In person | Participant | No | In−person demonstration or training | Community participation (village health committees and community volunteers) in leading and developing 7-day HIVST campaign | Routine facility-based HIV testing |
| **Jamil 2017** | Facility-based | HCW/ Researcher | Health facility/ Research Unit | In person | Participant | No | Instruction enhancement, video or study hotline | Supported confirmatory testing and counselling for HIV +ve | Routine facility-based HIV testing |
| **Katz 2018** | Facility-based | HCW/ Researcher | Health facility/ Research Unit | In person | Participant | No | In−person demonstration or training | Pre- and post-test counseling materials, a list of local HIV-related resources and condoms | Routine facility- based HIV testing |
| **Kelvin 2018** | Facility-based | HCW/ Researcher | Roadside wellness clinics at transit hubs | In person | Participant or assisted by HCW | Yes: Routine test / supervised or unsupervised HIVST | In−person observation or supervision | Pre and post-test counselling (phone); remuneration for completing baseline and post-test questionnaires | Routine provider administered rapid HIVT |
| **Kelvin 2019a** | Facility-based | HCW/ Researcher | Roadside wellness clinics at transit hubs | In person | Participant or assisted by HCW | Yes: Routine test / supervised or unsupervised HIVST | In−person observation or supervision | Text messages weekly x 3 informing of HIVST availability; post-test counselling and follow-up of those taking kit home | Routine facility-based HIV testing |
| **Kelvin 2019b** | Facility-based | HCW/ Researcher | Roadside wellness clinics at transit hubs | In person | Participant or assisted by HCW | Yes: Routine test / supervised or unsupervised HIVST | In−person observation or supervision | Text messages weekly x 3 informing of HIVST availability; post-test counselling and follow-up of those taking kit home | Routine facility-based HIV testing |
| **Lightfoot 2018** | Community-based | Peers | In community door-to-door | In person | Participant or assisted by peer | No | In−person demonstration or training | Incentives for survey completion, in the form of gift card or cash. Pay-for-performance to peers for each test distributed. | Routine facility-based HIVT testing |
| **MacGowan 2019** | Online & mail | Website | NA | Mail | Participant | No | Instruction enhancement, video or study hotline | Phone counselor & mental health counselling; online instructional video; remuneration for study activities | Website with routine HIV testing information |
| **Masters 2016** | Secondary | HCW to ANC client to partner | Facility and Community/ home | In person | Participant | No | Instruction enhancement, video or study hotline | Advise on partner negotiation and communication | Invitation card to partner to attend health services for HIV testing |
| **Merchant 2018** | Online & mail | Website/ Study app | NA | Mail | Participant | No | No or basic support | Remuneration for survey completion | Routine facility-based HIV testing |
| **Mulubwa 2019** | Community-based* | HCW | In community door-to-door | In person | Participant | Yes: Supervised, unsupervised, and secondary distribution option | In−person observation or supervision | Additional 7 day follow-up of those who had unsupervised self-tests or 2ndary distribution | Door-to-door routine HIV testing |
| **Nguyen 2019** | Community-based | Peers | In community door-to-door | In person | Participant or assisted by peer | Yes: Routine test / HIVST | In−person demonstration or training | Referral and follow-up visits conducted for reactive clients to encourage linkage to care. Partner notification services provided to all confirmed cases. | Door-to-door routine HIV testing |
| **Ortblad 2017** | Facility & secondary by peer | HCW or peer | Facility and Community/ home | In person | Participant | No | Group demonstration | 2 HIVST arms: HIVST coupon for facility testing or HIVST delivered to participants by peer | Routine facility-based HIV testing |
| **Pai 2018** | Facility-based | HCW/ Researcher | Health facility/ Research Unit | In person | Participant or assisted by HCW | Yes: Supervised, unsupervised | In−person observation or supervision | 2 HIVST arms: take home unsupervised HIVST or clinic based supervised HIVST | Routine facility-based HIV testing |
| **Patel 2019** | Facility-based | HCW/ Researcher | Emergency room | In person | Participant | No | No or basic support | Results reported on internet-based STIs/HIV testing recruitment. Also received five referral cards to give to their peers. | Routine HIV testing information pamphlet |
| **Pettifor 2018** | Facility-based* | HCW/ Researcher | Health facility/ Research Unit | In person | Participant | Yes: Routine test / HIVST | No or basis support | Women were given 5 invitations to test for free at local clinics. | Routine facility-based HIV testing |
| **Phanuphak 2019** | Online | Website/ study app | NA | Mail | Participant | Yes: Routine test / Online couselling + Routine test / HIVST | Online with virtual real−time supervision | Live chat application used for supervision | Online or in-person pre-test counseling and routine facility-based HIV testing |
| **Qin 2017** | Online, community-based, facility or secondary by peer | Website, HCW or peers | NA, facility, community, or home | Mail or in person | Participant or assisted by HCW | No | No or basic support | Post -test counseling received by three quarters of participants, either online, or in person, or over the phone. | Routine facility-based HIV testing |
| **Rich 2018** | Online & mail | Website | NA | Mail | Participant | Yes: Routine test / supervised or unsupervised HIVST | Instruction enhancement, video or telephone support | Text messages x reminding clients to report results. If no results reported, phone call attempted by peer. | Routine facility-based HIV testing |
| **Stafylis 2018** | Community-based | Vending machines | Commercial sex venues | Vending machine | Participant | No | No or basic support | Each kit contained additional information about local medical assistance in case of a reactive test result, hotline number, and invitation flyer to secure online survey. | Routine facility-based HIV testing |
| **Tang 2018** | Online & mail | Social networking mobile phone app | NA | Mail | Participant | No | Instruction enhancement, video or study hotline | Crowdsourcing community mobilization initiative in study cities for intervention design. Additional HIV self-testing promotional text messages | Routine facility-based HIV testing |
| **Tsamwa 2018** | Community-based | Community volunteers | In community door-to-door and from distributor home | In person | Participant | No | In−person demonstration or training | CBDAs visit clients after distributing kit to enquire whether the kit has been used, collect the sealed questionnaire and used kit, and provide advice on referral to additional care if the client discloses a re-active HIVST result. Clients also drop used kits in locked boxes within each community | Routine facility-based HIV testing |
| **Van der Elst 2017** | Community-based | Peers | In community | In person | Participant or assisted by peer | No | In-person observation or supervision | Participants asked to report for confirmatory testing at fixed-site clinic, irrespective of HIVST result | Routine facility-based HIV testing |
| **Wang 2017** | Online & mail | HCW/ Researcher | NA | Mail | Participant | No | Online promotional video with virtual real−time supervision | Health promotion videos; remuneration for survey completion; live chat application used for supervision | Routine facility-based HIV testing with health promotion video |
| **Wray 2018** | Online & mail | Website/ Study app | NA | Mail | Participant | No | No or basic support | Monitoring of kit opening with associated counselor follow up in one arm; remuneration for study activities | Letter with routine HIV testing information |

## S2(a)Table: Risk of bias for included studies - RCT

| Study | Sequence generation | Allocation concealment | Blinding of personnel and participants | Blinding of outcome assessment | Attrition bias | Selective reporting | Other bias | Cluster - recruitment bias | Cluster baseline imbalance | Cluster: loss of clusters | Cluster incorrect analysis | Overall ROB |
| --- | --- | --- | --- | --- | --- | --- | --- | --- | --- | --- | --- | --- |
| Chanda 2017 | Low | Low | High | Unclear | Low | Low | Low | Low | Low | Low | Low | High |
| Choko 2019a | Low | Low | High | High | Low | Low | Low | High | Low | Low | Low | High |
| Choko 2019b | Low | Low | High | High | Unclear | Low | Low | High | Low | Unclear | Unclear | High |
| Dovel 2018 | Low | Unclear | High | High | Low | Unclear | Low | High | Unclear | Low | Low | High |
| Dovel 2019 | Unclear | Unclear | High | High | Low | Low | Unclear |  |  |  |  | High |
| Gichangi 2018 | Low | Low | High | High | Low | Low | Low |  |  |  |  | High |
| Indravudh 2018 | Low | Low | High | High | Low | Low | Low | Low | Unclear | Low | Low | High |
| Indravudh 2019 | Low | Low | High | High | Unclear | Low | Low | Low | Unclear | Low | Low | High |
| Jamil 2017 | Low | Low | High | Low | Low | Low | Low |  |  |  |  | High |
| Katz 2018 | Low | Low | High | Unclear | Low | Low | Low |  |  |  |  | High |
| Kelvin 2018 | Unclear | Low | High | Low | Low | Low | Low |  |  |  |  | High |
| Kelvin 2019a | Unclear | Low | High | Unclear | Low | Low | Low |  |  |  |  | High |
| Kelvin 2019b | Unclear | Low | High | Unclear | Low | Low | Low |  |  |  |  | High |
| MacGowan 2019 | Low | Low | High | High | High | Low | Unclear |  |  |  |  | High |
| Masters 2016 | Low | Low | High | High | Low | Low | Low |  |  |  |  | High |
| Merchant 2018 | Low | Unclear | High | High | High | Low | Low |  |  |  |  | High |
| Mulubwa 2019 | Low | Low | High | Low | Low | Low | Low | Low | Low | Low | Low | High |
| Ortblad 2017 | Low | Low | High | High | Low | Low | Low | Low | Low | Low | Low | High |
| Patel 2018 | Unclear | Low | High | High | High | Low | Low |  |  |  |  | High |
| Pettifor 2018 | Unclear | Unclear | High | Unclear | Low | Unclear | Low |  |  |  |  | High |
| Tang 2018 | Low | Unclear | High | Low | Low | Low | Low | Low | Low | Low | Low | High |
| Tsamwa 2018 | Low | Unclear | High | High | Low | Low | Unclear | Low | Low | Low | Low | High |
| Wang 2017 | Low | Low | High | Low | Low | Low | Low |  |  |  |  | High |
| Wray 2018 | Low | Low | High | High | Low | Low | Unclear |  |  |  |  | High |

## S2(b)Table: Risk of bias for included studies – Observational studies

|  | Selection of cohorts | | | Comparability | | | Outcome | | | Total | | |
| --- | --- | --- | --- | --- | --- | --- | --- | --- | --- | --- | --- | --- |
| Study | Representativeness of exposed cohort | Selection of non-exposed cohort | Ascertainment of exposure | | Demonstration that outcome of interest was not present at start of study | Cohorts comparable | | Assessment of outcome | Length of follow-up | | Loss to follow-up rate | Overall Quality |
| Green 2018 |  |  | - | |  | - | |  | - | | - | Poor |
| Lightfoot 2018 |  | - |  | | - |  | | - |  | | - | Poor |
| Nguyen 2019 |  |  | - | | - | - | | - | - | |  | Poor |
| Pai 2018 |  | - | - | | - | - | | - | - | | - | Poor |
| Phanuphak 2019 |  |  |  | |  | - | |  |  | | - | Good |
| Qin 2017 |  |  | - | |  | - | | - |  | |  | Fair |
| Rich 2018 |  | - | - | | - | - | | - |  | | - | Poor |
| Stafylis 2018 |  |  |  | | - | - | | - | - | | - | Poor |
| VanDerElst 2017 |  |  |  | | - | - | | - |  | |  | Fair |

## S3(a)Table: HIV testing uptake among all randomized or enrolled - outcomes extracted from contributing studies

| **Author Year** | **Study arm** | **Outcome** | **Time point** | **Offered HIV test** | **Tested for HIV** | **aRR** | **Lower CI** | **Upper CI** |
| --- | --- | --- | --- | --- | --- | --- | --- | --- |
| Choko 2019a* | Standard of care | HIV testing uptake | 28 days | 408 | 71 |  |  |  |
| Choko 2019a* | Partner community distribution HIVST | HIV testing uptake | 28 days | 1941 | 1801 |  |  |  |
| Choko 2019b(i)* | Partner community distribution HIVST | HIV testing uptake | 28 days | 169 | 101 |  |  |  |
| Choko 2019b(i)* | Standard of care | HIV testing uptake | 28 days | 234 | 81 |  |  |  |
| Choko 2019b(ii)* | Partner community distribution HIVST | HIV testing uptake | 28 days | 1469 | 989 |  |  |  |
| Choko 2019b(ii)* | Standard of care | HIV testing uptake | 28 days | 1396 | 515 |  |  |  |
| Dovel 2018* | Standard of care | HIV testing uptake | 1 day | 3788 | 509 |  |  |  |
| Dovel 2018* | HCW facility distribution HIVST | HIV testing uptake | 1 day | 2097 | 1063 |  |  |  |
| Dovel 2019 | Partner community distribution HIVST | HIV testing uptake | 4 weeks | 349 | 282 |  |  |  |
| Dovel 2019 | Standard of care | HIV testing uptake | 4 weeks | 135 | 39 |  |  |  |
| Gichangi 2018 | Partner community distribution HIVST | HIV testing uptake | 3 months | 938 | 239 |  |  |  |
| Gichangi 2018 | Standard of care | HIV testing uptake | 3 months | 472 | 322 |  |  |  |
| Jamil 2017 | Standard of care | HIV testing uptake | 12 months | 182 | 170 |  |  |  |
| Jamil 2017 | HCW facility distribution HIVST | HIV testing uptake | 12 months | 180 | 122 |  |  |  |
| Katz 2018 | Standard of care | HIV testing uptake | 15 months | 116 | 96 |  |  |  |
| Katz 2018 | HCW facility distribution HIVST | HIV testing uptake | 15 months | 114 | 92 |  |  |  |
| Kelvin 2018 | HIVST (delivery, choice, coupon) | HIV testing uptake | 3 days | 155 | 113 |  |  |  |
| Kelvin 2018 | Standard of care | HIV testing uptake | 3 days | 150 | 131 |  |  |  |
| Kelvin 2019a | HCW facility distribution HIVST | HIV testing uptake | 2 months | 1510 | 20 |  |  |  |
| Kelvin 2019a | Standard of care | HIV testing uptake | 2 months | 750 | 31 |  |  |  |
| Kelvin 2019b | HCW facility distribution HIVST | HIV testing uptake | 2 months | 1446 | 89 |  |  |  |
| Kelvin 2019b | Standard of care | HIV testing uptake | 2 months | 750 | 119 |  |  |  |
| MacGowan 2019 | Standard of care | HIV testing uptake | Multiple | 1325 | 969 |  |  |  |
| MacGowan 2019 | Online & mail distribution | HIV testing uptake | Multiple | 1340 | 619 |  |  |  |
| Masters 2016 | Partner community distribution HIVST | HIV testing uptake | 3 months | 297 | 258 |  |  |  |
| Masters 2016 | Standard of care | HIV testing uptake | 3 months | 303 | 148 |  |  |  |
| Merchant 2018 | Standard of care | HIV testing uptake | 3 months | 141 | 79 |  |  |  |
| Merchant 2018 | Online & mail distribution | HIV testing uptake | 3 months | 142 | 94 |  |  |  |
| Ortblad 2017(i) | HCW community distribution HIVST | HIV testing uptake | 4 months | 336 | 288 |  |  |  |
| Ortblad 2017(i) | Standard of care | HIV testing uptake | 4 months | 328 | 263 |  |  |  |
| Ortblad 2017(ii) | Standard of care | HIV testing uptake | 4 months | 328 | 263 |  |  |  |
| Ortblad 2017(ii) | Peer community distribution HIVST | HIV testing uptake | 4 months | 296 | 261 |  |  |  |
| Ortblad 2017(iii) | Peer community distribution HIVST | HIV testing uptake | 4 months | 296 | 261 |  |  |  |
| Ortblad 2017(iii) | HCW community distribution HIVST | HIV testing uptake | 4 months | 336 | 288 |  |  |  |
| Patel 2019 | HCW facility distribution HIVST | HIV testing uptake | 1 month | 48 | 2 |  |  |  |
| Patel 2019 | Standard of care | HIV testing uptake | 1 month | 52 | 14 |  |  |  |
| Pettifor 2018 | HCW facility distribution HIVST | HIV testing uptake | 3 months | 144 | 60 |  |  |  |
| Pettifor 2018 | Standard of care | HIV testing uptake | 3 months | 140 | 117 |  |  |  |
| Wang 2017 | Standard of care | HIV testing uptake | 6 months | 215 | 193 |  |  |  |
| Wang 2017 | Online & mail distribution | HIV testing uptake | 6 months | 215 | 109 |  |  |  |
| Wray 2018 | Online & mail distribution | HIV testing uptake | 7 months | 43 | 43 |  |  |  |
| Wray 2018 | Standard of care | HIV testing uptake | 7 months | 22 | 16 |  |  |  |
| Indravudh 2018 | HCW community distribution HIVST vs standard of care | HIV testing uptake | 12 months |  |  | 1.33 | 1.12 | 1.59 |
| Tang 2018 | Online & mail distribution vs standard of care | HIV testing uptake | 3 months |  |  | 1.43 | 1.19 | 1.73 |
| Tsamwa 2018 | HCW community distribution HIVST vs standard of care | HIV testing uptake | 12 months |  |  | 1.08 | 0.94 | 1.24 |
| Mulubwa 2019 | HCW community distribution HIVST vs HCW community TT | HIV testing uptake | 3 months |  |  | 1.04 | 0.99 | 1.07 |
| Chanda 2017(ii) | Peer vs standard of care | HIV testing uptake | 4 months |  |  | 1.11 | 0.98 | 1.27 |
| Chanda 2017(i) | Coupon vs standard of care | HIV testing uptake | 4 months |  |  | 1.06 | 0.92 | 1.22 |
| Chanda 2017(iii) | Peer distribution vs coupon | HIV testing uptake | 4 months |  |  | 1.05 | 0.94 | 1.18 |
| Indravudh 2019 | HCW community distribution HIVST vs standard of care | HIV testing uptake | 3 months |  |  | 2 | 1.8 | 2.21 |

aRR: adjusted risk ratio; CI: confidence interval; * further adjustment for clustering conducted using design effect as detailed in methods; Standard of care = HCW facility based routine HIV testing; there were no observational studies that provided a denominator for inclusion in the analysis of uptake.

## S3(b) Table: HIV positivity among HIV tested – outcomes extracted from contributing studies

| **Author Year** | **Study arm** | **Outcome** | **Time point** | **HIV positive** | **HIV tested** | **aRR** | **Lower CI** | **Upper CI** |
| --- | --- | --- | --- | --- | --- | --- | --- | --- |
| Choko 2019a* | Standard of care | All positive | 28 days | 3 | 71 |  |  |  |
| Choko 2019a* | Partner community distribution HIVST | All positive | 28 days | 43 | 1801 |  |  |  |
| Choko 2019b(i)* | Partner community distribution HIVST | All positive | 28 days | 46 | 225 |  |  |  |
| Choko 2019b(i)* | Standard of care | All positive | 28 days | 9 | 81 |  |  |  |
| Choko 2019b(ii)* | Partner community distribution HIVST | All positive | 28 days | 20 | 2096 |  |  |  |
| Choko 2019b(ii)* | Standard of care | All positive | 28 days | 1 | 515 |  |  |  |
| Dovel 2018* | Standard of care | All positive | 1 day | 6 | 261 |  |  |  |
| Dovel 2018* | HCW facility distribution HIVST | All positive | 1 day | 27 | 1063 |  |  |  |
| Dovel 2019 | Partner community distribution HIVST | All positive | 4 weeks | 28 | 135 |  |  |  |
| Dovel 2019 | Standard of care | All positive | 4 weeks | 4 | 18 |  |  |  |
| Green 2018 | HCW community distribution TT | Confirmed HIV positive | December 2015 to September 2017 | 1386 | 23180 |  |  |  |
| Green 2018 | HCW community distribution HIVST | Confirmed HIV positive | May 2016 to September 2017 | 269 | 4220 |  |  |  |
| Jamil 2017 | Standard of care | All positive | 12 months | 0 | 122 |  |  |  |
| Jamil 2017 | HCW facility distribution HIVST | All positive | 12 months | 3 | 170 |  |  |  |
| Katz 2018 | Standard of care | All positive | 15 months | 2 | 92 |  |  |  |
| Katz 2018 | HCW facility distribution HIVST | All positive | 15 months | 3 | 96 |  |  |  |
| Kelvin 2018 | HIVST (delivery, choice, coupon) | All positive | 3 days | 0 | 150 |  |  |  |
| Kelvin 2018 | Standard of care | All positive | 3 days | 2 | 155 |  |  |  |
| Kelvin 2019a | HCW facility distribution HIVST | HIV positivity (EMR) | 2 months | 5 | 26 |  |  |  |
| Kelvin 2019a | Standard of care | HIV positivity (EMR) | 2 months | 0 | 10 |  |  |  |
| Kelvin 2019b | HCW facility distribution HIVST | HIV positivity (EMR) | 2 months | 5 | 119 |  |  |  |
| Kelvin 2019b | Standard of care | HIV positivity (EMR) | 2 months | 0 | 43 |  |  |  |
| Lightfoot 2018 | HCW community distribution TT | All positive | 15 months | 18 | 1205 |  |  |  |
| Lightfoot 2018 | Peer community distribution HIVST | All positive | 15 months | 7 | 114 |  |  |  |
| MacGowan 2019 | Standard of care | All positive | 12 months | 11 | 619 |  |  |  |
| MacGowan 2019 | Online & mail distribution | All positive | 12 months | 25 | 969 |  |  |  |
| Masters 2016 | Partner community distribution HIVST | All positive | 3 months | 2 | 258 |  |  |  |
| Masters 2016 | Standard of care | All positive | 3 months | 4 | 148 |  |  |  |
| Merchant 2018 | Standard of care | All positive | 3 months | 1 | 81 |  |  |  |
| Merchant 2018 | Online & mail distribution | All positive | 3 months | 1 | 98 |  |  |  |
| Mulubwa 2019 | HCW community distribution TT | Confirmed HIV positive | 3 months | 204 | 7800 |  |  |  |
| Mulubwa 2019 | HCW community distribution HIVST | Confirmed HIV positive | 3 months | 199 | 8077 |  |  |  |
| Nguyen 2019 | Peer community distribution HIVST | Confirmed HIV positive | Not described | 63 | 891 |  |  |  |
| Nguyen 2019 | Peer community distribution TT | Confirmed HIV positive | Not described | 167 | 3087 |  |  |  |
| Nichols 2019* | HCW community distribution HIVST | All positive | 6 months | 56 | 5353 |  |  |  |
| Nichols 2019* | Standard of care | All positive | 6 months | 214 | 6728 |  |  |  |
| Ortblad 2017(i) | HCW community distribution HIVST | All positive | 4 months | 80 | 289 |  |  |  |
| Ortblad 2017(i) | Standard of care | All positive | 4 months | 53 | 294 |  |  |  |
| Ortblad 2017(ii) | Standard of care | All positive | 4 months | 53 | 294 |  |  |  |
| Ortblad 2017(ii) | Peer community distribution HIVST | All positive | 4 months | 44 | 260 |  |  |  |
| Ortblad 2017(iii) | Peer community distribution HIVST | All positive | 4 months | 44 | 260 |  |  |  |
| Ortblad 2017(iii) | HCW community distribution HIVST | All positive | 4 months | 80 | 289 |  |  |  |
| Pai 2018 | HCW facility distribution HIVST | All positive | Not described | 120 | 1250 |  |  |  |
| Pai 2018 | Standard of care | All positive | Not described | 80 | 1250 |  |  |  |
| Phanuphak 2018 | Standard of care | All positive | 6 months | 3 | 356 |  |  |  |
| Phanuphak 2018 | Online & mail distribution | All positive | 6 months | 4 | 208 |  |  |  |
| Qin 2017 | Standard of care | Confirmed HIV positive | Not described | 15 | 306 |  |  |  |
| Qin 2017 | Online & mail distribution | Confirmed HIV positive | Not described | 24 | 341 |  |  |  |
| Rich 2018 | Online & mail distribution | All positive | 28 days | 5 | 498 |  |  |  |
| Rich 2018 | Standard of care | All positive | 28 days | 13 | 4120 |  |  |  |
| Stafylis 2018 | HCW community distribution TT | All positive | Not described | 10 | 1024 |  |  |  |
| Stafylis 2018 | Vending machine distribution HIVST | All positive | Not described | 11 | 945 |  |  |  |
| VanDerElst 2017 | Standard of care | Confirmed HIV positive | Not described | 24 | 690 |  |  |  |
| VanDerElst 2017 | Peer community distribution HIVST | Confirmed HIV positive | Not described | 29 | 337 |  |  |  |
| Wang 2017 | Standard of care | All positive | 6 months | 0 | 109 |  |  |  |
| Wang 2017 | Online & mail distribution | All positive | 6 months | 1 | 193 |  |  |  |
| Wray 2018 | Online & mail distribution | All positive | 7 months | 0 | 43 |  |  |  |
| Wray 2018 | Standard of care | All positive | 7 months | 0 | 16 |  |  |  |
| Chanda 2017(iii) | Peer distribution vs coupon | All positive | 4 months |  |  | 1.01 | 0.72 | 1.39 |
| Chanda 2017(i) | Coupon vs standard of care | All positive | 4 months |  |  | 0.92 | 0.65 | 1.28 |
| Chanda 2017(ii) | Peer vs standard of care | All positive | 4 months |  |  | 0.91 | 0.66 | 1.27 |

aRR: adjusted risk ratio; CI: confidence interval; * further adjustment for clustering conducted using design effect as detailed in methods; Standard of care = HCW facility based routine HIV testing; ART initiation selected preferentially as outcome, linkage to care used if ART initiation not available.

## S3(c) Table: Linkage to ART or HIV care among HIV positive: outcomes extracted from contributing studies

| **Author Year** | **Study arm** | **Outcome** | **Time point** | **Linked** | **HIV positive** | **aRR** | **Lower CI** | **Upper CI** |
| --- | --- | --- | --- | --- | --- | --- | --- | --- |
| Choko 2019a* | Standard of care | ART initiation | 28 days | 3 | 3 |  |  |  |
| Choko 2019a* | Partner community distribution HIVST | ART initiation | 28 days | 39 | 43 |  |  |  |
| Choko 2019b(i)* | Partner community distribution HIVST | Linked to HIV care | 28 days | 46 | 46 |  |  |  |
| Choko 2019b(i)* | Standard of care | Linked to HIV care | 28 days | 9 | 9 |  |  |  |
| Choko 2019b(ii)* | Partner community distribution HIVST | Linked to HIV care | 28 days | 20 | 20 |  |  |  |
| Choko 2019b(ii)* | Standard of care | Linked to HIV care | 28 days | 1 | 1 |  |  |  |
| Dovel 2018* | Standard of care | ART initiation | 3 months | 19 | 27 |  |  |  |
| Dovel 2018* | HCW facility distribution HIVST | ART initiation | 3 months | 5 | 6 |  |  |  |
| Dovel 2019 | Partner community distribution HIVST | ART initiation | 6 months | 3 | 4 |  |  |  |
| Dovel 2019 | Standard of care | ART initiation | 6 months | 7 | 30 |  |  |  |
| Green 2018 | HCW community distribution TT | Linked to HIV care | December 2015 to September 2017 | 1290 | 1386 |  |  |  |
| Green 2018 | HCW community distribution HIVST | Linked to HIV care | May 2016 to September 2017 | 243 | 269 |  |  |  |
| Jamil 2017 | Standard of care | Linked to HIV care | 12 months | 0 | 0 |  |  |  |
| Jamil 2017 | HCW facility distribution HIVST | Linked to HIV care | 12 months | 3 | 3 |  |  |  |
| Katz 2018 | Standard of care | Linked to HIV care | 15 months | 2 | 2 |  |  |  |
| Katz 2018 | HCW facility distribution HIVST | Linked to HIV care | 15 months | 3 | 3 |  |  |  |
| MacGowan 2019 | Standard of care | Linked to HIV care | 12 months | 16 | 25 |  |  |  |
| MacGowan 2019 | Online & mail distribution | Linked to HIV care | 12 months | 10 | 11 |  |  |  |
| Masters 2016 | Partner community distribution HIVST | Linked to HIV care | 3 months | 2 | 8 |  |  |  |
| Masters 2016 | Standard of care | Linked to HIV care | 3 months | 3 | 4 |  |  |  |
| Nguyen 2019 | Peer community distribution HIVST | ART initiation | Not described | 59 | 63 |  |  |  |
| Nguyen 2019 | Peer community distribution TT | ART initiation | Not described | 156 | 167 |  |  |  |
| Ortblad 2017(i) | HCW community distribution HIVST | ART initiation | 4 months | 27 | 80 |  |  |  |
| Ortblad 2017(i) | Standard of care | ART initiation | 4 months | 24 | 53 |  |  |  |
| Ortblad 2017(ii) | Standard of care | ART initiation | 4 months | 24 | 53 |  |  |  |
| Ortblad 2017(ii) | Peer community distribution HIVST | ART initiation | 4 months | 19 | 44 |  |  |  |
| Ortblad 2017(iii) | Peer community distribution HIVST | ART initiation | 4 months | 19 | 44 |  |  |  |
| Ortblad 2017(iii) | HCW community distribution HIVST | ART initiation | 4 months | 27 | 80 |  |  |  |
| Rich 2018 | Online & mail distribution | Linked to HIV care | 28 days | 12 | 13 |  |  |  |
| Rich 2018 | Standard of care | Linked to HIV care | 28 days | 4 | 5 |  |  |  |
| Stafylis 2018 | HCW community distribution TT | Linked to HIV care | Not described | 10 | 10 |  |  |  |
| Stafylis 2018 | Vending machine distribution HIVST | Linked to HIV care | Not described | 7 | 11 |  |  |  |
| VanDerElst 2017 | Standard of care | ART initiation | 1 Day(s) | 24 | 29 |  |  |  |
| VanDerElst 2017 | Peer community distribution HIVST | ART initiation | 14 Day(s) | 20 | 24 |  |  |  |
| Wang 2017 | Standard of care | Linked to HIV care | 6 months | 1 | 1 |  |  |  |
| Wang 2017 | Online & mail distribution | Linked to HIV care | 6 months | 0 | 0 |  |  |  |
| Chanda 2017(i) | HCW facility HIVST vs standard of care | ART initiation | 4 months |  |  | 0.77 | 0.56 | 1.11 |
| Chanda 2017(ii) | Peer community HIVST vs standard of care | ART initiation | 5 months |  |  | 0.89 | 0.6 | 1.31 |
| Chanda 2017(iii) | Peer community HIVST vs HCW facility HIVST | ART initiation | 6 months |  |  | 0.88 | 0.66 | 1.18 |
| Tsamwa 2018 | HCW community distribution HIVST | ART initiation | 12 months |  |  | 0.96 | 0.76 | 1.21 |

aRR: adjusted risk ratio; CI: confidence interval; * further adjustment for clustering conducted using design effect as detailed in methods; Standard of care = HCW facility based routine HIV testing; ART initiation selected preferentially as outcome, linkage to care used if ART initiation not available.

## S4 Table: Sub-Saharan Africa network meta-analysis relative effects (league) table of HIVST distribution strategies - HIV testing uptake

|  | **HCW_community_HIVST** | **HCW_community_TT** | **HCW_facility_HIVST** | **HCW_facility_TT** | **Partner_community_HIVST** | **Peer_community_HIVST** |
| --- | --- | --- | --- | --- | --- | --- |
| **HCW_community_HIVST** | **HCW_community_HIVST** | 0.96 (0.38, 2.43) | 1.21 (0.63, 2.27) | 0.7 (0.41, 1.2) | 1.71 (0.88, 3.32) | 1 (0.48, 2.09) |
| **HCW_community_TT** | 1.04 (0.41, 2.64) | **HCW_community_TT** | 1.25 (0.41, 3.94) | 0.73 (0.25, 2.18) | 1.78 (0.55, 5.64) | 1.04 (0.32, 3.43) |
| **HCW_facility_HIVST** | 0.83 (0.44, 1.59) | 0.8 (0.25, 2.44) | **HCW_facility_HIVST** | 0.58 (0.41, 0.81) | 1.42 (0.84, 2.4) | 0.83 (0.5, 1.37) |
| **HCW_facility_TT** | 1.43 (0.83, 2.43) | 1.38 (0.46, 4.02) | 1.71 (1.23, 2.44) | **HCW_facility_TT** | 2.43 (1.63, 3.64) | 1.42 (0.87, 2.36) |
| **Partner_community_HIVST** | 0.59 (0.3, 1.14) | 0.56 (0.18, 1.81) | 0.7 (0.42, 1.19) | 0.41 (0.28, 0.61) | **Partner_community_HIVST** | 0.59 (0.31, 1.12) |
| **Peer_community_HIVST** | 1 (0.48, 2.07) | 0.96 (0.29, 3.11) | 1.2 (0.73, 1.99) | 0.7 (0.42, 1.15) | 1.71 (0.89, 3.18) | **Peer_community_HIVST** |

HCW: health care worker; TT: traditional HIV testing (rapid finger prick test conducted by HCW)

##

## S5 Table: Direct pairwise meta-analyses of effects of HIVST distribution strategies on HIV testing uptake

| **Sub-Saharan Africa** | | | | |
| --- | --- | --- | --- | --- |
| **Strategy 1** | **Strategy 2** | **Pooled Risk Ratio** | **Pooled Risk Difference** | **Studies** |
| Partner-community-HIVST | HCW-facility-TT | 2.45 (95% CI: 1.87-3.21) | 0.90 (95%CI:0.63-1.00) | Choko 2019b, Masters 2016, Choko 2019b, Gichangi 2018, Dovel 2019, Choko 2019a |
| HCW-facility-HIVST | HCW-FB-TT | 1.78 (95%CI:1.36-2.32) | 0.57 (95%CI: 0.31-0.84) | Chanda 2017, Ortblad 2017, Kelvin 2018, Pettifor 2018, Kelvin 2019a, Kelvin 2019b, Dovel 2018 |
| Peer-community-HIVST | HCW-facility-TT | 1.09 (95%CI: 1.03 -1.14) | 0.08 (95%CI: 0.03-0.13) | Chanda 2017, Ortblad 2017 |
| Peer-community-HIVST | HCW-facility-HIVST | 1.07 (95%CI: 0.98-1.18) | 0.07 (95%CI:0.00-0.16) | Chanda 2017, Ortblad 2017 |
| HCW-community-HIVST | HCW-facility-TT | 1.43 (95%CI: 0.95 -2.13) | 0.35 (95%CI: -0.05-0.76) | Tsamwa 2018, Indravudh 2018, Indravudh 2019 |
| HCW-community-HIVST | HCW-community-TT | 1.03 (95%CI: 1 -1.07) | 0.03 (95%CI: 0-0.07) | Mulubwa 2019 |
| **North America, Asia and Pacific region** | | | | |
| **Strategy 1** | **Strategy 2** | **Pooled Risk Ratio** | **Pooled Risk Difference** | **Studies** |
| HCW-facility-HIVST | HCW-facility-TT | 1.29 (95%CI: 0.93-1.78) | 0.25 (95%CI: -0.07-0.58) | Katz 2018, Jamil 2017, Patel 2018 |
| Online-mail-HIVST vs | HCW-Facility-TT | 1.48 (95%CI: 1.31-1.68) | 0.39(95%CI: 0.27-0.52) | Merchant 2018, Wray 2018, MacGowan 2017, Wang 2017, Tang 2018 |

HCW: health care worker; TT: traditional HIV testing (rapid finger prick test conducted by HCW

## S6 Table: Sub-Saharan Africa network meta-analysis relative effects (league) table, metaregression (by population type) - HIV testing uptake

|  | **HCW_community_TT** | **HCW_community_HIVST** | **HCW_facility_TT** | **HCW_facility_HIVST** | **Partner_community_HIVST** | **Peer_community_HIVST** |
| --- | --- | --- | --- | --- | --- | --- |
| **HCW_community_TT** | **HCW_community_TT** | 1.03 (0.34, 3.21) | 0.76 (0.15, 2.34) | 1.47 (0.11, 4.09) | 1.43 (0.52, 21.61) | 1.2 (0.05, 3.45) |
| **HCW_community_HIVST** | 0.97 (0.31, 2.96) | **HCW_community_HIVST** | 0.74 (0.27, 1.24) | 1.43 (0.18, 2.55) | 1.38 (0.76, 12.75) | 1.17 (0.08, 2.28) |
| **HCW_facility_TT** | 1.31 (0.43, 6.48) | 1.36 (0.81, 3.64) | **HCW_facility_TT** | 1.94 (0.49, 2.68) | 1.82 (1.19, 21.58) | 1.59 (0.22, 2.51) |
| **HCW_facility_HIVST** | 0.68 (0.24, 8.91) | 0.7 (0.39, 5.68) | 0.52 (0.37, 2.02) | **HCW_facility_HIVST** | 0.93 (0.51, 37.96) | 0.81 (0.32, 1.3) |
| **Partner_community_HIVST** | 0.7 (0.05, 1.94) | 0.73 (0.08, 1.32) | 0.55 (0.05, 0.84) | 1.08 (0.03, 1.97) | **Partner_community_HIVST** | 0.88 (0.01, 1.78) |
| **Peer_community_HIVST** | 0.84 (0.29, 18.27) | 0.86 (0.44, 11.87) | 0.63 (0.4, 4.55) | 1.23 (0.77, 3.13) | 1.14 (0.56, 79.7) | **Peer_community_HIVST** |

This analysis is adjusted for strategy population target – men, women or both groups. aRR: adjusted risk ratio; CI: confidence interval; * further adjustment for clustering conducted using design effect as detailed in methods; Standard of care = HCW facility based routine HIV testing; ART initiation selected preferentially as outcome, linkage to care used if ART initiation not available.

## S7(a) League table: Sub-Saharan Africa network meta-analysis – sensitivity analysis (FSW excluded), HIV testing uptake

|  | **HCW_community_TT** | **HCW_community_HIVST** | **HCW_facility_TT** | **HCW_facility_HIVST** | **Partner_community_HIVST** |
| --- | --- | --- | --- | --- | --- |
| **HCW_community_TT** | **HCW_community_TT** | 1.03 (0.39, 2.82) | 0.73 (0.23, 2.27) | 1.53 (0.45, 5.28) | 1.73 (0.52, 5.84) |
| **HCW_community_HIVST** | 0.97 (0.36, 2.56) | **HCW_community_HIVST** | 0.7 (0.4, 1.22) | 1.48 (0.72, 3.1) | 1.67 (0.83, 3.46) |
| **HCW_facility_TT** | 1.38 (0.44, 4.42) | 1.42 (0.82, 2.52) | **HCW_facility_TT** | 2.12 (1.35, 3.34) | 2.39 (1.59, 3.64) |
| **HCW_facility_HIVST** | 0.65 (0.19, 2.24) | 0.68 (0.32, 1.39) | 0.47 (0.3, 0.74) | **HCW_facility_HIVST** | 1.13 (0.62, 2.06) |
| **Partner_community_HIVST** | 0.58 (0.17, 1.93) | 0.6 (0.29, 1.21) | 0.42 (0.28, 0.63) | 0.88 (0.49, 1.62) | **Partner_community_HIVST** |

HCW: health care worker; TT: traditional HIV testing (rapid finger prick test conducted by HCW); Chanda 2017; Ortblad 2017 and Kelvin 2019b

## S7(b) Network meta-analysis ranking probabilities: Sub-Saharan Africa network meta-analysis – sensitivity analysis (FSW excluded), HIV testing uptake

| **Testing and distribution strategy** | **Probability of ranking 1** | **Probability of ranking 2** | **Probability of ranking 3** | **Probability of ranking 4** | **Probability of ranking 5** |
| --- | --- | --- | --- | --- | --- |
| **HCW_community_TT** | 0.13 | 0.11 | 0.24 | 0.26 | 0.26 |
| **HCW_community_HIVST** | 0.02 | 0.08 | 0.46 | 0.40 | 0.04 |
| **HCW_facility_TT** | 0.00 | 0.00 | 0.06 | 0.24 | 0.69 |
| **HCW_facility_HIVST** | 0.27 | 0.49 | 0.15 | 0.07 | 0.00 |
| **Partner_community_HIVST** | 0.57 | 0.31 | 0.08 | 0.03 | 0.00 |

HCW: health care worker; TT: traditional HIV testing (rapid finger prick test conducted by HCW)

## S8 Table: North America; Asia & Pacific region network meta-analysis relative effects (league) table – HIV testing uptake

|  | **HCW_facility_HIVST** | **HCW_facility_TT** | **Online_mail_HIVST** |
| --- | --- | --- | --- |
| **HCW_facility_HIVST** | **HCW_facility_HIVST** | 0.7 (0.29, 1.12) | 1.1 (0.42, 2.19) |
| **HCW_facility_TT** | 1.42 (0.89, 3.51) | **HCW_facility_TT** | 1.55 (1.01, 2.76) |
| **Online_mail_HIVST** | 0.91 (0.46, 2.39) | 0.64 (0.36, 0.99) | **Online_mail_HIVST** |

HCW: health care worker; TT: traditional HIV testing (rapid finger prick test conducted by HCW)

## S1 Appendix: Supplementary methods

### Implementation strategy definitions:

- Who delivered the intervention
  - *Peer:* Defined as someone who shares the lived experience of the individual patient, in the included studies in this review peers were exclusively used for HIVST distribution among sex-workers and MSM, peers were sex workers and MSM respectively.
  - *Partner:* sexual partners (for studies in this review this included partners of HIV positive patients in care and partners of antenatal clinic attendees
  - *Health care worker:* includes both health care workers with formal training – such as nurses and physicians and ‘lay health care workers’ in sub-Saharan Africa (a cadre of health staff who do not have formal training in health but receive specific training to e.g. perform HIV testing or provide HIV adherence counselling).
- How the intervention was delivered
  - This is linked to who delivered the intervention in most cases as delivery was person to person, but in some cases HIVST were delivered through
  - vending machines, or
  - by ordering the HIVST online and having it delivered in the mail
- Where the intervention was delivered
  - At the health facility (includes any level of academic or primary care clinic, as well as researcher and NGO run HIV clinics), this includes peers delivering coupons/vouchers to people to collect an HIVST at a health facility
  - In the community – any location outside of the health facility and within the community (includes homes, social, and sex work venues)

### Outcome definitions:

1. Uptake of HIV testing at the longest time point among all randomized or enrolled
2. HIV positivity among those tested for HIV: confirmed HIV positive test, where not available observed or self-reported HIV test result used
3. Linkage to ART or any care among HIV positive: ART initiation selected primarily, in studies that did not include details of ART initiation, this includes linkage to any HIV services.

### Pairwise meta-analyses methods:

- We used the meta-package in R programming software and generic inverse variance to conduct pairwise meta-analyses. For individually randomized studies we incorporated numerators and denominators to generate effect estimates. For cluster randomized trials we preferentially included cluster adjusted estimates from trials but in cases where these data were not sufficient for our analyses we used the methods detailed in the Cochrane handbook for adjusting by the design effect ^(1)^:
- **“**The effective sample size of a single intervention group in a cluster-randomized trial is its original sample size divided by a quantity called the ‘design effect’. The design effect is

1 + (M – 1) ICC,

where M is the average cluster size and ICC is the intracluster correlation coefficient. A common design effect is usually assumed across intervention groups. For dichotomous data both the number of participants and the number experiencing the event should be divided by the same design effect.”

- For cohort studies we preferentially included effect estimates adjusted for baseline imbalances, however no studies provided the adjusted effect estimates required for analyses, we therefore incorporated unadjusted numerators and denominators and did not pool cohort and RCT findings.
- We used random effect models for all analyses.

### Network meta-analyses methods:

- Network meta-analyses allow both direct and indirect comparisons between treatment strategies (where there is no direct comparison or if there is no direct comparison between treatment groups).
- Both frequentist and Bayesian hierarchical meta-analysis models were considered for network meta-analysis methods ^(2)^, and in order to generate ranking probabilities, we used Bayesian Hierarchical models with Markov chain Monte Carlo (MCMC) simulations, which, given that no prior information was entered into the model, approximates results from frequentist approaches ^(3)^. For distributions commonly used in statistics, the area under the distribution curve can be obtained with an integral for­mula. However, with the Bayesian method, it is difficult to calcu­late it if the posterior distribution does not follow a commonly used distribution. In this case, the Markov chain Monte Carlo (MCMC) simulation can be used to calculate it reversely. We applied this approach to generate network estimates.
- We generated network diagrams, used random effects models and evaluated convergence by adjusting the number of chains appropriate for multi-chain, the number of data for removal of initial effect (burn-in), the number of iterations, and the extraction interval (thin).
- We generated two networks, as we considered setting to be an effect modifier (which would violate the assumption of intransitivity) and additionally conducted meta-regression and sensitivity analysis to explore the effects of population subgroups on network estimates ^(1)^.
- We evaluated inconsistency between direct and indirect comparisons (this was only relevant to the peer -community distribution comparisons) using the node-splitting technique.
- Ranks are based on the results of each MCMC cycle, where testing strategies are ranked according to the estimated effect size, the proportion of the cycles in which a strategy ranks first out of the total gives the probability that the strategy ranks first among all strategies. Ranking probabilities are similarly generated for being second best, third best, and so on, and probabilities sum to one for each treatment and each rank ^(4)^.

**References**

1. Higgins JPT, Thomas J, J. C, M. C, Li T, Page MJ, et al. Cochrane Handbook for Systematic Reviews of Interventions. version 6.0 ed: Cochrane; 2019.

2. Hoaglin DC, Hawkins N, Jansen JP, Scott DA, Itzler R, Cappelleri JC, et al. Conducting indirect-treatment-comparison and network-meta-analysis studies: report of the ISPOR Task Force on Indirect Treatment Comparisons Good Research Practices: part 2. Value in health : the journal of the International Society for Pharmacoeconomics and Outcomes Research. 2011;14(4):429-37.

3. Shim SR, Kim SJ, Lee J, Rucker G. Network meta-analysis: application and practice using R software. Epidemiology and health. 2019;41:e2019013.

4. Salanti G, Ades AE, Ioannidis JP. Graphical methods and numerical summaries for presenting results from multiple-treatment meta-analysis: an overview and tutorial. J Clin Epidemiol. 2011;64(2):163-71.
